# The Treatment of Pericementitis

**Published:** 1894-07

**Authors:** C. N. Johnson


ARTICLE V.

### THE TREATMENT OF PERICEMENTITIS.

BY C. N. JOHNSON.

Probably one of the most painful affections with which the dentist has to deal is that of acute pericementitis immediately preceding alveolar abscess. This is easily accounted for when we consider the anatomical relations of the tissues surrounding the root of a tooth. The pericementum is encased in a bony socket with unyielding walls on either side, and a slight degree of inflammation results in an undue amount of pressure. From whatever causes pericementitits may be induced there sometimes comes a stage when no kind of treatment directed into the pulp chamber and through the canal is the least effective. It is to the consideration of this phase of the affection that I wish briefly to call attention.

In those cases where the canals have been opened up and a free vent made for the escape of gases, and this has failed to bring relief, it is an indication that the tissues of the peridental membrane have become so far implicated that the inflammatory process will go on independent of further irritation. This has always proved in my hands the most difficult stage of the affection to control, till within the past two or three years. During that time I have followed a practice which, if faithfully carried out, has seldom failed to give satisfactory results. The treatment consists in the application of moist heat to the gums surrounding the affected tooth.

The method of procedure is as follows: Water as hot as can be borne is taken up in a large bulb syringe having a fine point. A jet is thrown on the gums and into the cavity of the tooth, all cotton or other dressings having previously been removed from the canals. The tooth and surrounding tissues are thus submerged. During the refilling of the syringe the water is to be retained in the mouth and then emptied into the spittoon just previous to another application. The water should be kept on a gas stove near at hand, and the heat gradually raised as the tissues will admit. It will be found that in the end water extremely hot may be used if the heat be gradually increased and one of the essentials to success in this treatment is to employ an exceedingly high degree of heat. If the tongue and other tissues of the mouth remote from the region of the tooth are too much affected by the water, a saliva ejector may be used to carry off the water after it has flown over the gum in the immediate vicinity of the inflammation. The gum in this region will often tolerate a much higher temperature than the normal tissues. In fact, an application so hot as to be extremely painful to normal tissue often proves instantly soothing to the affected parts. The process should be kept up till perfect relief has been obtained, and the length of time necessary for this varies in different cases. In some instances where the pain is most distressing prior to the application the sense of relief will appear so suddenly as to prove astonishing, while in others it will require persistent treatment for perhaps thirty or even forty minutes before a substantial effect is produced. I have never yet encountered a case where persistent effort failed to bring a cessation of pain. As to the permanency of the relief, this is governed largely by conditions and circumstances.

If the general tone of the system is bad; if there is universal congestion strongly marked by the symptoms of what is commonly called "a heavy cold;" if the circulatory and absorbent systems are badly out of condition; or if the excretory organs fail to perform their function, then the relief is necessarily temporary, and we must resort to constitutional treatment in order to gain permanent results.

A patient in this condition should be given a rapidly acting cathartic, the citrate of magnesia in large doses having proved in my experience the most pleasant and satisfactory. Again, if the inflaammation has gone on to a point where suppuration has begun in the apical space, we cannot hope for complete relief till the abscess has pointed and discharged, but even in these cases we may be reasonably sure of preventing extensive infiltration and puffing of the superficial tissues, and also of mitigating the suffering.

As to the circumstances which may render the relief temporary, I have noted that if the patient left the office immediately after the hot water application, and went into the open air on a cold day without carefully protecting the whole side of the face from the air, the pain would ordinarily recur. In other words, exposure would bring back the trouble. But not the least among the gratifying results that have followed this line of practice in my hands is the large per cent of cases where a permanent relief has been gained.

A criticism might be made by some as to the advisability of leaving the cavity and canals open during the treatment. The argument might be used that there was danger of additional infection through the medium of the open canals, but I have never seen a case where I could trace any evil effects to this cause. My reasons for leaving the canals open are, that I wish the hot water to reach the apical space if possible, and that there may be a likelihood of the water, in flooding the cavity and canals, floating to the surface or dislodging small particles of debris that may have been packed in the canals near the apical foramen, and which the broach has failed to bring away. I never leave the cavity open when I dismiss the patient. If the tooth is not too sensitive to pressure I usually dry the canals as thoroughly as possible after the pain is relieved, and then flood them with an antiseptic. A small pledget of cotton saturated with the antiseptic is then placed loosely in the chamber, and the cavity sealed with Gilbert's temporary stopping.

The canals themselves are never packed with cotton at this sitting, and if the tooth is much raised in its socket, and extremely sensitive to pressure, I do not even attempt to seal with gutta-percha. A tooth in this condition should be worked on with instruments as little as possible. The mechanical irritation invariably results in increased sensitiveness.

When the tooth is loosened and sore from swelling of the pericementum, I simply dry the canals, flood them with an antiseptic, and place some cotton in the cavity merely to keep food and debris from packing into the tooth. I should much prefer dismissing the patient in this condition and taking chances of infection through the imperfectly sealed cavity to attempting a thorough sealing at this sitting. These are mostly emergency sittings, and our principal office is to relieve the present distress. Usually in twenty-four hours the tooth will be in a condition to work on, and it may then be treated and sealed.

The simplicity of the hot water treatment would seem to argue that the case could as well be treated by the patient in his own home as by the dentist in his office, but my experience has taught me that the results are seldom satisfactory. Somehow patients do not succeed, no matter how careful the instructions. They do not use the water hot enough, or they do not apply it properly, or do not keep up the application a sufficient length of time.

In fact, the element of time in this treatment proves one of the greatest obstacles to its practical application. As has been stated these are emergency cases, and are likely to call for attention at any hour through the day without previous appointment. In a full practice where almost every moment of every hour is systematically assigned in advance for the regular routine work of the office, it is exceedingly trying to the operator to be called away from his work to devote any considerable time to an unexpected patient. In those cases where I have fallen short of producing the most satisfactory results it has been traceable to this dilemma. And yet a dentist's first duty as a practitioner and as an an individual is to relieve suffering no matter when or how it appears.

In searching for a theory to account for the results obtained by the application of hot water I was led to consult Dr. W. T. Belfield on the subject. I then learned for the first time that he had long been a strong advocate of hot applications for the relief of pain in other tissues. His theory was that heat, especially moist heat, was an anæsthetic. This would account for the relief of pain temporarily, but if this were the only action of hot water the pain would recur in a short period. To account for the permanent results which I claim to have undoubtedly secured in many cases, he said that hot water applied in the manner I had indicated would prove a very rapid and effectual absorbent. It is to this lattter quality that I attribute the lasting results, and this argues especially for a somewhat protracted application.

Dr. Belfield also stated that in the use of hot water, a very minute stream should be used to play on the tissues. A jet such as would come from a hypodermic needle he had found more effective than a larger stream.

In conclusion, I may add that in some cases where the pain has been excruciating, I have added to the hot water a few drops of carbolic acid, making about a five per cent solution. The anesthetic effect of carbolic acid is thus added to that of the hot water, and the relief is exceedingly prompt and very gratifying to the patient. Hot water is also excellent for after pain and hemorrhage.—*Review.*

